# Measuring stigma after spinal cord injury: Development and psychometric characteristics of the SCI-QOL Stigma item bank and short form

**DOI:** 10.1179/1079026815Z.000000000410

**Published:** 2015-05

**Authors:** Pamela A. Kisala, David S. Tulsky, Natalie Pace, David Victorson, Seung W. Choi, Allen W. Heinemann

**Affiliations:** 1 Department of Physical Therapy, University of Delaware, College of Health Sciences, Newark, DE, USA; 2 Kessler Foundation Research Center, West Orange, NJ, USA; 3 Northwestern University Feinberg School of Medicine, Chicago, IL, USA; 4 McGraw-Hill Education CTB, Monterey, CA, USA; 5 Rehabilitation Institute of Chicago, Chicago, IL, USA

**Keywords:** Health-Related Quality of Life, Outcomes Assessment (Healthcare), Patient Reported Outcomes, Social Stigma, Spinal Cord Injuries

## Abstract

**Objective:**

To develop a calibrated item bank and computer adaptive test (CAT) to assess the effects of stigma on health-related quality of life in individuals with spinal cord injury (SCI).

**Design:**

Grounded-theory based qualitative item development methods, large-scale item calibration field testing, confirmatory factor analysis, and item response theory (IRT)-based psychometric analyses.

**Setting:**

Five SCI Model System centers and one Department of Veterans Affairs medical center in the United States.

**Participants:**

Adults with traumatic SCI.

**Main Outcome Measures:**

SCI-QOL Stigma Item Bank

**Results:**

A sample of 611 individuals with traumatic SCI completed 30 items assessing SCI-related stigma. After 7 items were iteratively removed, factor analyses confirmed a unidimensional pool of items. Graded Response Model IRT analyses were used to estimate slopes and thresholds for the final 23 items.

**Conclusions:**

The SCI-QOL Stigma item bank is unique not only in the assessment of SCI-related stigma but also in the inclusion of individuals with SCI in all phases of its development. Use of confirmatory factor analytic and IRT methods provide flexibility and precision of measurement. The item bank may be administered as a CAT or as a 10-item fixed-length short form and can be used for research and clinical applications.

## Introduction

Spinal cord injury (SCI) involves damage to the spinal cord from external trauma^1^ such as a motor vehicle crash, fall, or gunshot wound. Most individuals who experience an SCI lose some level of function in their limbs, resulting in dependence on mobility aids – most commonly, manual or power wheelchairs.^2^ Frequent secondary complications of SCI include bowel, bladder, and sexual dysfunction,^2^ chronic pain, fatigue, and mood disturbance.^3–5^ Social participation, or the ability to be involved and engaged in life situations^6^ such as those related to family and friends, vocation, and leisure activities – may be quite restricted. Given the visible consequences of SCI, the experience of stigma in social settings can be profound and may have detrimental effects on individuals’ health-related quality of life (HRQOL).^4,7–9^

Individuals with physical disabilities such as SCI are a minority group and stand out from the population because of their physical limitations.^8^ Susman^10^ describes the relationship between stigma and disability in detail, emphasizing that disabling conditions are stigmatizing to the extent that they elicit negative responses. SCI-related stigma is a result of the visibility of the condition as well as the degree that a person is dependent on help. When communicating in group settings, wheelchair-using individuals often express feelings of being dismissed as if their physical disability is also a mental disability, and report being spoken to as though they cannot comprehend.^7,11^ These findings were confirmed in unpublished focus group data described by Tulsky *et al.*,^12^ wherein participants with SCI described feeling invisible yet conspicuous at the same time and also reported being spoken to slowly, as if their wheelchair was the sign of a cognitive impairment. These examples are the epitome of stigma.

The concept of stigma, signifying a mark of shame or discredit both from one's own internalized attributions, as well as from the perceptions of others, has a long ancestry in the social sciences. Erving Goffman proposed that people are stigmatized when they possess an attribute that is considered undesirable and ‘deeply discrediting.’ Goffman's work identified three aspects of stigma: character blemishes, features of identity (e.g. race, religion, sexual preference) and physical ‘deformities’ such as disability.^13^ The societal response to such attributes may include labeling, stereotyping, and/or discrimination.^14^ Though Goffman's seminal work was published over 60 years ago, it is almost universally referenced in modern publications on health- or disability-related stigma.

Several theories surrounding the course of stigma exist in the literature. Goffman suggested that an individual is stigmatized by society when others can see the disability^13^ and all visible disabilities or undesirable physical characteristics are certain to be stigmatized. In contrast, Schneider postulated that the state of being stigmatized was dependent on whether the individual chose to see him/herself in that way.^15^ These two views compliment a more recent conceptualization in which stigma development involves three distinctions: enacted stigma, perceived (or felt) stigma, and internalized stigma.^16–18^ Enacted stigma refers to episodes of discrimination and misconduct (e.g. stereotyping, labeling) towards someone who is in a stigmatized group. Perceived stigma describes one's awareness of enacted stigma and can create isolation or exclusion from normal activities, while internalized stigma implies agreement with enacted stigma and a sense of shame from it.^19^

Health-related stigma is based on negative characteristics of a health condition or state.^20^ In the last several decades, there has been a great deal of research on the impact of stigma on individuals with conditions such as HIV/AIDS, cancer, mental illness and epilepsy. For example, a recent study examining the effects of stigma on individuals with epilepsy found perceived stigma to negatively impact physical, mental and social health outcomes.^14^ Unfortunately, though cross-cultural studies suggest that the consequences of stigma are similar across health conditions,^20^ data on the impact of stigma on individuals with SCI are sorely lacking.

Knowledge on the effects of stigma on individuals with SCI is hindered by the lack of available patient-reported outcomes (PRO) measures of stigma for use in individuals with SCI. The Short Form Health Survey (SF-36),^21^ Sickness Impact Profile (SIP), Life Satisfaction Questionnaire (LISAT-9/-1), Satisfaction with Life Scale (SWLS),^22^ and the 26-item version of the World Health Organization Quality of Life Scale (WHOQOL-BREF)^23^ have demonstrated reliability and validity when used in individuals with SCI, but have been developed with the general population and contain items across a wide range of physical, emotional, and social health. While useful, these instruments do not address the myriad of elements unique to SCI, and, most importantly, none assess stigma specifically.

There also exist a variety of measures intended to assess the objective existence and extent of stigma towards individuals with various types of disabilities including, most notably, the Scale of Attitudes Toward Disabled Persons.^24^ However, many would argue that it is the subjective experience of stigma that can affect HRQOL. As such, it is important to assess SCI-related stigma from the individual with SCI's own perspective. There are numerous scales measuring the subjective experience of stigma in individuals with a variety of chronic health conditions such as mental illness (e.g. Perceived Devaluation and Discrimination Scale,^25^ Internalized Stigma of Mental Illness,^26^ Self-stigma of Mental Illness Scale),^27^ HIV/AIDS (e.g. HIV Stigma Scale),^28^ and epilepsy (e.g. Stigma Scale of Epilepsy).^29^ These scales do focus on many aspects of stigma (e.g. perceived discrimination, enacted discrimination, alienation) that would seem to have face validity for individuals with SCI, however none have been used in SCI research. Therefore, their psychometric properties in individuals with SCI cannot be determined.

A recent National Institutes of Neurological Disorders and Stroke (NINDS) sponsored initiative (Neuro-QOL) developed a calibrated item bank of stigma in individuals with neurological disorders (i.e. stroke, epilepsy, multiple sclerosis, amyotrophic lateral sclerosis, and Parkinson's disease).^30,31^ However, individuals with SCI were not included in the development, calibration, or validation of this item bank, and most neurological disorders do not share the same level of visibility and cultural image of SCI. A primary goal of the spinal cord injury quality of life (SCI-QOL) initiative has been to adapt the Neuro-QOL for an SCI population. In the first phase of the SCI-QOL study, individuals with SCI cited SCI-related stigma as having a detrimental effect on HRQOL.^12^ Thus, the research team prioritized the development of an optimized version of the Neuro-QOL Stigma item bank for use in individuals with SCI. This manuscript details the development and calibration of the SCI-QOL Stigma item bank.

## Methods

### Item development

The first phase of the project included development of new PRO items and evaluation of existing Neuro-QOL items for use in individuals with SCI.^32^ A variety of qualitative methods were used to identify the most salient aspects of SCI-related stigma.

#### Individual interviews

A series of individual semi-structured interviews were conducted with 44 individuals with SCI.^33^ Participants were placed in the role of ‘expert’ and asked to list topics or issues that were important to address when assessing HRQOL in individuals with SCI. Participants discussed issues related to stigma such as being treated differently or unfairly because of their disability, not feeling accepted in social settings, and feeling pitied by others. Twenty-two items were written based on these comments.

#### Focus groups

The overarching themes that arose from the individual interviews served as a basis for more in-depth examination of physical-medical, emotional, and social components of HRQOL through a series of focus groups with community-dwelling individuals with SCI (12 groups; *n *= 65) and SCI clinicians (4 groups; *n *= 42) which were led by an experienced moderator (DV).^12^ Groups with individuals with SCI focused on one major HRQOL domain, either physical-medical, emotional, or social health, while provider groups covered all domains. Participants in all groups were asked open-ended questions about the definition of HRQOL and the ways in which an SCI could impact HRQOL. Participants were also asked to review and provide feedback on existing HRQOL domains and subdomains. A detailed grounded-theory based qualitative analysis of focus group transcripts was conducted^34^ and within the broader domain of social health and participation, 10% of consumer comments and 6% of clinician comments were related to stigma. These comments were then reworded as 12 PRO items to form the initial basis of the SCI-QOL Stigma item pool. The project team made a decision to include only those items related to enacted and perceived stigma, or the way an individual believes they are being perceived or treated by *others*, in the new measure of SCI-related stigma. In contrast, items related to internalized stigma, or one's perception of *oneself*, were included in the Self-Esteem item bank^35^ which assesses myriad aspects of an individual's self-appraisal.

#### Inclusion of Neuro-QOL items

One of the primary aims of the SCI-QOL project was to optimize the Neuro-QOL measurement system for individuals with SCI. Thus, all Neuro-QOL stigma items were reviewed for content and potential inclusion in the SCI-QOL Stigma bank. The Neuro-QOL items that captured perceived or enacted stigma (e.g. ‘Because of my illness, strangers tended to stare at me’) were adapted and reworded to fit an SCI population. Conversely, Neuro-QOL Stigma items related more to internalized Stigma or self-appraisal (e.g. ‘I was unhappy about how my illness affected my appearance’) were moved to the Self-Esteem bank. Individuals with SCI are not sick and do not have an ‘illness,’ so all Neuro-QOL Stigma items containing the phrase ‘because of my illness’ were modified with permission to ‘because of my injury’ for use in the SCI-QOL. The preliminary pool of SCI-QOL Stigma items was largely based on the Neuro-QOL Stigma bank, with 24 verbatim items included. In every case of a ‘new’ (i.e. derived from focus groups or individual interviews) item with similar content to a Neuro-QOL item, the Neuro-QOL item was retained and the new item discarded. The Neuro-QOL Stigma domain definition^36^ was adapted for an SCI population as follows: ‘Others’ perceptions of oneself and publically enacted negativity, prejudice, and discrimination as a result of injury-related manifestations.’

### Item refinement

A thorough qualitative item review^37,38^ was conducted on all ‘new’ (i.e. not from Neuro-QOL) items. Initially, project co-investigators with expertise in social and emotional issues related to SCI reviewed all of the ‘new’ items, making suggestions for revisions and deletions as appropriate. Items in the initial pool were revised to optimize wording, eliminate redundancies, and ensure consistency with the domain definition. All retained ‘new’ items were also rephrased for consistency with the Neuro-QOL Stigma item context of ‘Lately’ and the response set of ‘Never /Rarely /Sometimes /Often /Always.’ Next, a series of cognitive debriefing interviews^39^ was conducted with individuals with SCI (*n *= 5 per item).^33^ Participants were asked to rephrase items in their own words and to describe their decision-making and response retrieval processes. Once the study team incorporated cognitive interview feedback into the item pool, all ‘new’ items underwent a translatability review^40^ to flag wording that would be problematic to translate into Spanish. The last step to prepare the item pool for calibration testing was to conduct a reading level review using the Lexile Framework^41^ to ensure that all items were written at or below a 5^th^ grade reading level. A final pool of 30 items (24 Neuro-QOL items and 6 new items) was utilized for the calibration field testing phase of the project.

### Calibration field testing

#### Sample

Adults with traumatic SCI were recruited from 6 collaborating centers including 5 SCI Model Systems (SCIMS) centers (University of Michigan, Kessler Institute for Rehabilitation/Kessler Foundation, Rehabilitation Institute of Chicago, Craig Hospital, University of Washington) and one Department of Veterans’ Affairs (VA) center (the James J. Peters/Bronx VA). Care was taken to recruit a heterogeneous sample, balanced across diagnosis (paraplegia vs. tetraplegia), severity (complete vs. incomplete injury) and time since injury (<1 year, 1–3 years, >3 years). We did not stratify by gender since the SCI population is predominantly (i.e. approximately 79%) male.^42^ Inclusion criteria were traumatic etiology of injury, ability to speak and understand English, and age 18 years or older at the time of study participation. Level and etiology of injury and American Spinal Injury Association (ASIA) Impairment Scale (AIS)^43^ grade were documented through medical record review.

#### Procedure

The thirty Stigma items were administered with other SCI-QOL items in interview format by trained study personnel using a customized web-based administration platform. Response cards depicting the appropriate response set for each item bank were placed in front of the participant (if in person) or provided to the participant by mail and/or email (if via phone). Interviewers read the items from the computer screen aloud and recorded the participants’ responses which were uploaded in real time. The Institutional Review Board at each participating center reviewed and approved this study.

#### Analysis

There are two key assumptions underlying the successful application of IRT to any pool of items. First, the pool of items must be essentially unidimensional (i.e. assess a single dominant construct). Items within a unidimensional pool must also be locally independent, that is, the only factor affecting the response to any one item given the response to any other item is the level of the underlying trait in question.^44,45^ For this study, confirmatory factor analyses with MPlus version 6.0a were conducted to assess fit to a unidimensional model. Several indices of goodness-of-fit were considered. The Tucker-Lewis Index (TLI) is a non-normed fit index which adjusts for the number of degrees of freedom in the model. TLI values above 0.9 are considered good fit and values above 0.95 indicate excellent fit. The comparative fit index (CFI) is a normed fit index which compares the current model to a null or independent model. Possible CFI values range from 0.0 to 1.0, with values above 0.9 indicating good fit and values above 0.95 indicating excellent model fit. The root mean square error of approximation (RMSEA), which divides the *F* statistic by degrees of freedom to compensate for model complexity, was also used to assess fit to the unidimensional model. When interpreting the RMSEA, perfect fit would be indicated by a value of 0, with commonly accepted cutoff criteria of 0.08 for acceptable fit^46^ and 0.05 for excellent fit.^47^ To assess adherence to the IRT assumption of local independence, items were evaluated for local item dependence (LID). Item pairs exhibiting a residual correlation >|0.2| were flagged and at least one of the items was removed from the item pool.

Item slope (discrimination) and threshold (difficulty) parameters were estimated using the graded response IRT model (GRM).^48^ The S-X^2^ test using the IRTFIT^49^ macro program was used to further evaluate item fit, with P < 0.05 indicating poor fit and P < 0.01 necessitating item removal. Finally, differential item functioning (DIF) analyses were conducted using *lordif*^50^ to examine whether any included items exhibited bias towards any demographic or diagnostic subgroup. DIF analyses identify items that exhibit a difference in item response functions depending on injury subgroup or demography. The most important indicator of DIF is not whether items systematically differentiate relevant subgroups, but whether they do so even after controlling for the level of the underlying trait (e.g. stigma).^51^ DIF was examined for six categories: age (≤49 vs ≥50), sex (male vs female), education (some college and lower vs college degree and above), diagnosis (tetraplegia vs. paraplegia), severity (incomplete vs. complete), and time post injury (>1 year vs. <1 year). Items were flagged if the probability associated with the χ^2^ test <0.01 and McFadden's pseudo *R*^2^ effect size >0.02 (a small but non-negligible effect).

#### Transformation to Neuro-QOL metric

Once developed, the initial IRT parameters for the final bank of Stigma items underwent a linear transformation to the Neuro-QOL metric so that SCI-QOL Stigma scores reference the same general neurological population as do the Neuro-QOL Stigma scores. The linking procedure, described by Tulsky *et al.*,^33^ consisted of 6 steps. First, the linking configuration was determined through counts of calibration and anchor items Anchor and calibration parameters were then identified for matched items. Linking was conducted using the Stocking and Lord^52^ method. Scatter plots of item parameters and item response plots were created/examined for anchor items, transformation constants were estimated, and the initial item parameters were modified accordingly.

#### Short form development

For each SCI-QOL item bank, a short, fixed-length form has been developed. These ‘short forms’ provide a paper-and-pencil alternative to CAT administration. Project co-investigators considered both clinical relevance and psychometric item characteristics in the selection of short form items IRT parameters of slope (discrimination) and thresholds (difficulty) were examined. The most informative 1–2 items (i.e. those with the highest slopes) were chosen within each quintile of difficulty. Investigators then considered clinical relevance and similarity to other included items in deciding whether to retain those items selected for their psychometric characteristics, or whether to replace any of them with items with slightly lower slopes.

#### Reliability study

A reliability study has been conducted with individuals with traumatic SCI as a part of an ongoing effort to quantify the psychometric properties of the SCI-QOL item banks. Participants recruited from 4 SCI Model Systems rehabilitation centers completed the SCI-QOL Stigma CAT and short form at baseline and 1–2 weeks. To assess test-retest reliability, Pearson's *r* and the intraclass correlation coefficient, ICC(2,1),^53,54^ were calculated and a Bland-Altman plot was developed.

## Results

### Participant characteristics

Detailed demographic information on focus group participants (*n *= 65 individuals with SCI and 42 SCI clinicians)^12^ and on participants in the reliability study (*n *= 245 individuals with SCI)^33^ has been published and is not repeated here. A total of 611 participants with traumatic SCI completed the SCI-QOL stigma items as a part of the large-scale SCI-QOL calibration study. Demographic and injury-related information on the calibration sample is summarized in Table 1.

**Table 1:** Demographic and Injury Characteristics of Calibration Sample

Variable	Stigma Bank Calibration Sample, *N* = 611
Mean (SD), N (%)	
Age (years)	42.9 (15.5)
Age at injury (years)	36.2 (17.1)
Sex	
Male	475 (78%)
Female	136 (22%)
Ethnicity	
Hispanic	61 (10%)
Non-Hispanic	547 (90%)
Not reported	3 (1%)
Race	
Caucasian	445 (74%)
African-American	101 (17%)
Asian	6 (1%)
American Indian/Alaska Native or Native Hawaiian/Pacific Islander	5 (1%)
More than one race	6 (1%)
Other or Not reported	41(7%)
Time Since Injury	6.7 (8.7)
< 1 year post injury	137 (22%)
1–3 years post injury	188 (31%)
> 3 years post injury	286 (47%)
Diagnosis	
Paraplegia Complete	152 (25%)
Paraplegia Incomplete	115 (19%)
Tetraplegia Complete	129 (21%)
Tetraplegia Incomplete	211 (35%)

### Analysis

A total of 3 CFA iterations were run. Five items were removed following the first iteration due to sparse data (i.e. fewer than 4 responses) in one or more categories (3 items) or low item-total correlations (2 items). Two of the removed items exhibited a category inversion whereby the mean raw score for individuals selecting category 5 was lower than for individuals selecting category 4. Following the second iteration of CFA, two additional items were removed for LID.

CFA analyses confirmed fit to a unidimensional model. CFI for the final 23-item bank was 0.941, TLI was 0.935, and RMSEA was 0.088. Item loadings on the single factor were acceptable with *R*^2^ for 17 items > 0.4, *R*^2^ for 4 items between 0.3 and 0.4, and *R*^2^ for 2 items approaching 0.3 (0.269 and 0.0292, respectively). No item pairs exhibited LID. Descriptive statistics on each of the 23 final items are located in Table 2.

**Table 2:** SCI-QOL Stigma Bank: Descriptive Item Statistics

Item ID	Item Stem	Mean	SD	% at Min	% at Max
SQNQSTG01	Because of my injury, some people seemed uncomfortable with me.	2.06	1.030	37.5	2.0
**SQNQSTG03**	**Because of my injury, I felt emotionally distant from other people.**	**2.26**	**1.143**	**33.6**	**3.8**
**SQNQSTG04**	**Because of my injury, I felt left out of things.**	**2.61**	**1.227**	**25.0**	**6.5**
SQNQSTG07	Because of my injury, I felt embarrassed in social situations.	1.97	1.048	43.5	2.1
**SQNQSTG08**	**Because of my injury, people avoided looking at me.**	**1.72**	0.**904**	**53.0**	**0.8**
**SQNQSTG09**	**Because of my injury, strangers tended to stare at me.**	**2.29**	**1.184**	**32.9**	**6.2**
**SQNQSTG10**	**Because of my injury, I worried about other people's attitudes towards me.**	**1.87**	**1.067**	**50.1**	**2.8**
SQNQSTG12	I was unhappy about how my injury affected my appearance.	2.60	1.351	29.8	11.9
SQNQSTG13	Because of my injury, it was hard for me to stay neat and clean.	1.86	1.074	50.6	3.1
SQNQSTG14	Because of my injury, people tended to ignore my good points.	1.67	0.876	56.1	0.7
SQNQSTG15	Because of my injury, I worried that I was a burden to others.	2.61	1.265	26.4	9.7
**SQNQSTG16**	**I felt embarrassed about my injury.**	**1.87**	**1.110**	**51.9**	**3.4**
SQNQSTG17	I felt embarrassed because of my physical limitations.	2.24	1.200	36.5	6.2
**SQNQSTG19**	**Because of my injury, I felt different from others.**	**2.65**	**1.293**	**25.5**	**10.6**
SQNQSTG20	I tended to blame myself for my problems	2.43	1.369	36.4	11.6
SQNQSTG21	Some people acted as though it was my fault I have this injury.	1.68	1.045	62.7	2.5
SQNQSTG22	I avoided making new friends to avoid telling others about my injury.	1.43	0.869	74.8	1.6
SQNQSTG26	I lost friends by telling them that I have this injury.	1.28	0.732	83.6	0.8
**SQStigma_19**	**Because of my injury, I felt like other people were uncomfortable around me.**	**1.96**	**1.018**	**43.5**	**1.6**
**SQStigma_20**	**Because of my injury, I felt like other people felt pity for me.**	**2.33**	**1.126**	**30.8**	**3.9**
**SQStigma_21**	**Because of my injury, I felt uncomfortable when people stared at me.**	**1.96**	**1.148**	**48.3**	**4.7**
SQStigma_23	Because of my injury, I was discriminated against	1.41	0.840	75.4	1.8
SQStigma_26	Because of my injury, I felt that other people had low expectations of me.	2.18	1.123	37.6	2.8

Note: Context for all items was: ‘Lately’. Response set was: Never/Rarely/Sometimes/Often/Always.

Positively worded items were scored 1–5 and negatively worded items were scored 5–1.

**Bold text** indicates items selected for the short form 10a.

Items and parameters copyright © 2015 David Tulsky and Kessler Foundation. All Rights Reserved. Scales should be accessed and used through the corresponding author or www.assessmentcenter.net. Do not modify items without permission from the copyright holder.

The final 23-item bank has a Cronbach's alpha value of 0.936. Item-total correlations range from 0.39 to 0.72. All of the items had more than 25% of the sample selecting category 1 (‘Never’) and less than 12% selecting category 5 (‘Always’).

Graded response model IRT analyses yielded slope values for the 23 items ranging from 1.10 to 2.87, with thresholds ranging from −0.87 to 3.95. Measurement precision in the theta range between –0.5 and 2.4 was roughly equivalent to a classical reliability of 0.95 or better. The S-X^2^ model fit statistics indicated adequate or better model fit statistics for all but one item at α = 0.05 and all items at α = 0.01. 11 items were flagged for DIF in at least one category based on the chi-square test; however, when the effect size measures were examined, the DIF was negligible. The 18 retained Neuro-QOL items served as ‘anchors’ to conduct the transformation of item parameters to the Neuro-QOL metric. Following transformation, slope values for the 23 items ranged from 1.81 to 4.72 and thresholds ranged from –0.16 to 2.77 (see Table 3). Mean (SD) of the sample shifted from 49.82 (9.66) before transformation to 53.18 (6.69) after transformation.

**Table 3:** SCI-QOL Stigma Items and Item Bank Parameters

Item ID	Item Stem	Item Response Theory Calibration Statistics				
		Slope	Threshold 1	Threshold 2	Threshold 3	Threshold 4
SQNQSTG01	Because of my injury, some people seemed uncomfortable with me.	2.95021	0.11562	0.73655	1.49901	2.10619
**SQNQSTG03**	**Because of my injury, I felt emotionally distant from other people.**	**3.78196**	**0.07016**	**0.56597**	**1.15756**	**1.68487**
**SQNQSTG04**	**Because of my injury, I felt left out of things.**	**3.23231**	**-0.16456**	**0.29323**	**0.90336**	**1.57542**
SQNQSTG07	Because of my injury, I felt embarrassed in social situations.	3.75865	0.25616	0.76731	1.36188	1.87497
**SQNQSTG08**	**Because of my injury, people avoided looking at me.**	**2.89686**	**0.43741**	**1.04407**	**1.83807**	**2.44852**
**SQNQSTG09**	**Because of my injury, strangers tended to stare at me.**	**1.90333**	**−0.08836**	**0.63533**	**1.52363**	**2.05068**
**SQNQSTG10**	**Because of my injury, I worried about other people's attitudes towards me.**	**4.08905**	**0.38340**	**0.84388**	**1.35054**	**1.75631**
SQNQSTG12	I was unhappy about how my injury affected my appearance.	3.36831	**−**0.03711	0.32177	0.88132	1.29624
SQNQSTG13	Because of my injury, it was hard for me to stay neat and clean.	2.13556	0.38557	1.04580	1.71990	2.28391
SQNQSTG14	Because of my injury, people tended to ignore my good points.	2.67501	0.50865	1.13658	2.01991	2.62682
SQNQSTG15	Because of my injury, I worried that I was a burden to others.	3.70374	**−**0.10193	0.28829	0.94647	1.35067
**SQNQSTG16**	**I felt embarrassed about my injury.**	**4.71630**	**0.40783**	**0.79000**	**1.23361**	**1.60130**
SQNQSTG17	I felt embarrassed because of my physical limitations.	4.13681	0.13825	0.55125	1.14886	1.49004
**SQNQSTG19**	**Because of my injury, I felt different from others.**	**3.49320**	**−0.13273**	**0.29850**	**0.88562**	**1.32433**
SQNQSTG20	I tended to blame myself for my problems	2.14101	0.04552	0.49908	1.14301	1.54671
SQNQSTG21	Some people acted as though it was my fault I have this injury.	1.80640	0.72425	1.25409	1.96741	2.66945
SQNQSTG22	I avoided making new friends to avoid telling others about my injury.	3.05811	0.90982	1.33232	1.76115	2.14859
SQNQSTG26	I lost friends by telling them that I have this injury.	2.46291	1.25272	1.61572	2.15829	2.69387
**SQStigma_19**	**Because of my injury, I felt like other people were uncomfortable around me.**	**4.11823**	**0.26484**	**0.72665**	**1.42424**	**1.90562**
**SQStigma_20**	**Because of my injury, I felt like other people felt pity for me.**	**3.44921**	**−0.00471**	**0.45546**	**1.21298**	**1.73276**
**SQStigma_21**	**Because of my injury, I felt uncomfortable when people stared at me.**	**3.90122**	**0.34977**	**0.77633**	**1.31655**	**1.60276**
SQStigma_23	Because of my injury, I was discriminated against	1.88079	1.09196	1.70201	2.40753	2.76919
SQStigma_26	Because of my injury, I felt that other people had low expectations of me.	3.15973	0.13671	0.56602	1.29416	1.91788

Note: Context for all items was: "Lately’. Response set was : Never/Rarely/Sometimes/Often/Always.

Positively worded items were scored 1–5 and negatively worded items were scored 5–1.

**Bold text** indicates items selected for the short form 10a.

Items and parameters copyright © 2015 David Tulsky and Kessler Foundation. All Rights Reserved. Scales should be accessed and used through the corresponding author or www.assessmentcenter.net. Do not modify items without permission from the copyright holder.

**Figure 1:**
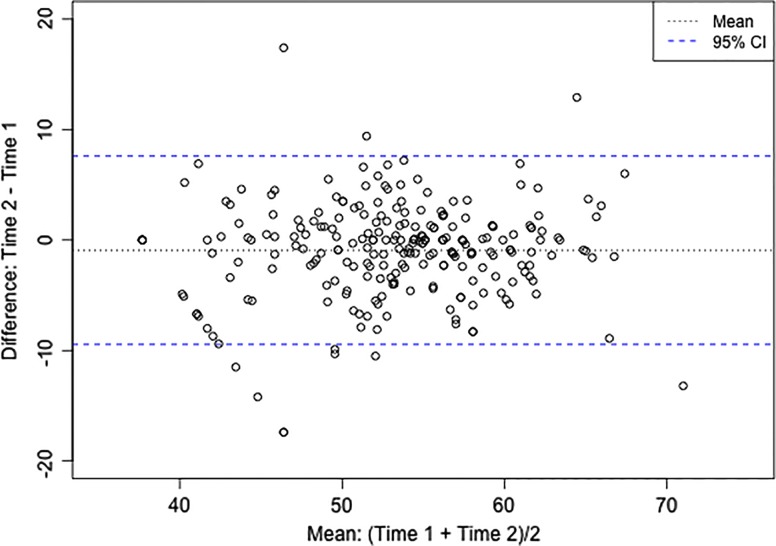
SCI-QOL Stigma: Bland-Altman Plot for 1–2 week test-retest.

The SCI-QOL Stigma bank demonstrates excellent reliability. Using the calibration data (*n *= 611), Cronbach's α = 0.936 for full bank administration and α = 0.895 for the 10-item short form. Furthermore, the correlation (Pearson's *r*) between the baseline and 1–2 week retest assessments was 0.80 for the CAT (*n *= 245; P < 0.001) and 0.84 for the 10-item short form (*n *= 168; P < 0.001). ICC (2,1) was 0.79 (95% CI: 0.74 to 0.84) and a Bland-Altman plot is provided as Fig. 1.

### Assessment center programming and short form item selection

The IRT parameters for each final SCI-QOL Stigma item were programmed into the Assessment Center^SM^^55^ platform, where the full bank, CAT, and a brief, fixed-length ‘short form’ are available free of charge (see www.assessmentcenter.net). A total of 10 items were selected for the initial SCI-QOL Stigma short form (SF). In keeping with the naming conventions of the Patient Reported Measurement Information System (PROMIS), this form is called the SCI-QOL v1.0 Stigma SF10a. Short form items are indicated by bold text in Tables 2 and 3.

**Figure 2:**
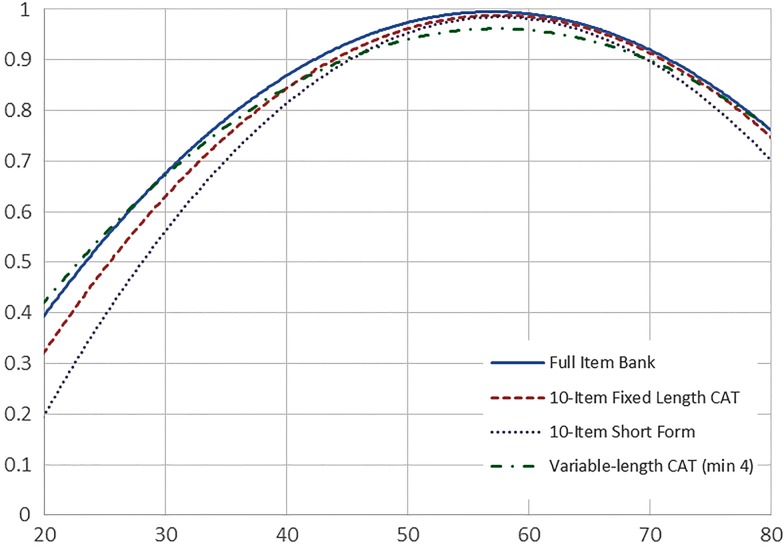
SCI-QOL Stigma: Measurement Reliability by Administration Option.

When administered as a CAT, by default Assessment Center will administer a minimum of 4 items and will continue to administer items until the standard error of measurement (SEM) falls below 0.3 or the maximum of 12 items is reached. Users may also modify these CAT parameters to ensure that participants complete a shorter or longer CAT. A comparison of the measurement precision of the full Stigma item bank, the Stigma CAT, and the Stigma SF10a can be found in Table 4. Furthermore, reliability curves for full bank, CATs of varying lengths, and SF are located in Fig. 2.

**Table 4:** Accuracy of Variable and Fixed-Length CAT and 10-item Short Form: Correlations with Full-bank score

Mode	N	# Items Admin				% Min	% Max	Corr. w/ Full Bank
		Mean	SD	Min	Max			
Variable-Length CAT (min 4)	611	5.3	2.6	4	12	72.3%	9.8%	0.95
Variable-Length CAT (min 8)	611	8.5	1.2	8	12	86.9%	9.8%	0.97
10-Item Fixed-Length CAT	611	10	0	10	10	n/a	n/a	0.98
10-Item Short Form	611	10	0	10	10	n/a	n/a	0.97

#### Scoring

Higher scores on the Stigma bank indicate a greater degree of perceived stigma. Though SCI-QOL scores were originally developed with a reference population of individuals with traumatic SCI, the transformation to the Neuro-QOL metric yields final standardized scores which now reference the mean of a broader population of individuals with neurological disorders. The Neuro-QOL normative sample for the Stigma item bank included 511 adults with stroke (*n* = 209), epilepsy (*n* = 183), Parkinson's disease (*n* = 50), multiple sclerosis (*n* = 84), and amyotrophic lateral sclerosis (*n* = 18) (diagnoses are not mutually exclusive).^37^ Consequently, SCI-QOL Stigma scores are directly comparable to Neuro-QOL Stigma scores. For CAT administration, Assessment Center automatically transforms IRT-based scaled scores (theta values) into standardized T-scores (mean 50, SD 10). The short form does, however, need to be scored manually. Scores should be produced only for individuals who have completed all 10 items, and are computed by summing the responses to each of the items The lookup table provided as Table 5 can then be used to transform the raw score to the corresponding IRT-based T-score. A comparison of the range of scores and standard errors for the full bank, CAT, and SF administrations is located in Table 6.

**Table 5:** T-score lookup table for SCI-QOL Stigma SF10a

Raw score	T-score	Standard error
10	37.8	5.7
11	43.4	3.7
12	45.3	3.4
13	47.1	2.8
14	48.3	2.6
15	49.5	2.3
16	50.4	2.1
17	51.3	2.0
18	52.0	1.9
19	52.7	1.8
20	53.4	1.8
21	54.1	1.8
22	54.7	1.7
23	55.3	1.7
24	55.9	1.7
25	56.4	1.7
26	57.0	1.7
27	57.6	1.7
28	58.2	1.7
29	58.7	1.7
30	59.3	1.7
31	59.8	1.7
32	60.4	1.7
33	61.0	1.7
34	61.5	1.7
35	62.1	1.7
36	62.7	1.7
37	63.3	1.7
38	63.9	1.7
39	64.5	1.7
40	65.1	1.7
41	65.8	1.8
42	66.4	1.8
43	67.2	1.9
44	68.0	2.0
45	68.8	2.1
46	69.9	2.3
47	71.0	2.5
48	72.4	2.8
49	74.0	3.1
50	77.3	4.1

**Table 6:** Breadth of Coverage for Variable Length CAT, Fixed Length CAT, 10-Item Short Form, and Full Item Bank

Mode	N	T Score				Standard Error	
		Mean±SD	Range	% Ceiling	% Floor	Mean ± SD	Range
Variable-Length CAT (min 4)	611	53.08 ± 6.65	36.54–69.88	0.16%	4.75%	0.25 ± 0.08	0.19–0.55
Variable-Length CAT (min 8)	611	53.11 ± 6.71	36.54–73.22	0.16%	4.75%	0.20 ± 0.09	0.14–0.55
10-Item Fixed-Length CAT	611	53.10 ± 6.81	36.77–72.56	0.16%	5.40%	0.20 ± 0.10	0.13–0.55
10-Item Short Form	611	52.96 ± 6.92	37.8–71.0	0.16%	8.51%	0.22 ± 0.12	0.14–0.57
Full Bank	611	53.18 ± 6.69	35.89–71.05	0.16%	4.26%	0.16 ± 0.09	0.10–0.54

## Discussion

Stigma, or societal negativity, prejudice, or discrimination is a significant HRQOL concern of many individuals with SCI. To date, there have not been not been any measures of Stigma targeted to individuals with SCI, and the SCI-QOL Stigma bank addresses an overlooked need in rehabilitation outcomes assessment for individuals with SCI. Though the Neuro-QOL developed a calibrated bank of Stigma items, these items were not developed or tested with individuals with SCI and furthermore contained the phrase ‘because of my illness’ which individuals with SCI reported as inappropriate and problematic to respond to. Several Neuro-QOL items did not perform well in an SCI population (e.g. ‘I felt embarrassed about my speech’) whereas other important issues (e.g. ‘I was discriminated against’) are not addressed by Neuro-QOL. Additionally, Neuro-QOL included items related to one's perception of oneself alongside items related to the perceptions of the attitudes and behaviors of others; the SCI-QOL has tried to improve upon this conceptualization given the highly visible nature of SCI. Items related to appraisals or perceptions of oneself have been omitted from the SCI-QOL Stigma bank and have instead been included in a new bank of SCI-QOL items specifically related to valuations of one's competence and self-worth, or Self-Esteem.^35^

Developing and revising the SCI-QOL Stigma items based largely on the input of individuals with SCI and SCI clinicians has helped to ensure that the final SCI-QOL Stigma bank is conceptually grounded to relevant and important aspects of Stigma for the SCI population. It is worth noting, however, that developing items based on feedback from individuals with SCI does not mean that these items are only appropriate for individuals with SCI or that the items themselves could not be equally relevant for other disability populations (e.g. traumatic brain injury, multiple sclerosis). Conducting the calibration testing solely with individuals with SCI, though, has optimized the relevance of CAT-selected items for individuals with SCI. Furthermore, utilizing a linear transformation to place the item calibrations on the Neuro-QOL metric facilitating instantaneous comparison with the larger population of individuals with neurological disorders. The combination of qualitative item development and refinement methods used in conjunction with advanced psychometrics puts the SCI-QOL Stigma item bank at the cutting edge of test development.

Future research should ascertain if this item bank could be used to detect individuals at risk for poor psychosocial adjustment or if the perception of stigma is a state which could be treated. The SCI-QOL Stigma bank is not meant to be used as a diagnostic tool but rather to help researchers and clinicians assess and understand the impact of SCI-related stigma on individuals’ HRQOL. The SCI-QOL Stigma CAT or short form could be included as an outcome variable in clinical trials or intervention research and may also be used clinically to better direct therapy and treatment.

### Study limitations and future directions

A potential challenge faced by the SCI-QOL project team was the difficulty of creating a unidimensional assessment tool – a prerequisite for IRT analyses and CAT programming – while accurately representing multiple facets (e.g. perceived, enacted) of a construct such as stigma. There was some precedent to including them in a single item bank as the Neuro-QoL team had found that the items conformed to a unidimensional model. The SCI-QOL project team also wanted to include the most important aspects of stigma and see if the items conformed to a unidimensional model. Another challenge is that Neuro-QOL items were reworded, changing ‘illness’ to ‘injury,’ and an assumption was made that they would be psychometrically equivalent. We also felt that it is important to allow users a way to compare the SCI-QOL Stigma to the Neuro-QoL Stigma score to allow comparison across neurologic conditions. Therefore, we transformed the SCI-QOL Stigma scores to the Neuro-QOL metric as we had done in other groups.^33^ Further work examining the possible effect of this wording change is important to do in future research to determine if the items are still measuring the same thing. Also, we had redefined our construct of Stigma and had removed some Neuro-QOL items related to how an individual feels about themselves (a construct we call Self Esteem),^35^ further testing to examine if the underlying construct between the Neuro-QOL Stigma and SCI-QOL Stigma has changed.

Other future directions include examination of responsiveness of the SCI-QOL Stigma item bank to change over time, development of clinically relevant classifications of SCI-QOL Stigma scores, and assessment of convergent and divergent validity with other measures.

## Conclusions

The SCI-QOL Stigma item bank is a psychometrically sound measurement tool which can reliably estimate HRQOL effects of SCI-related stigmatization in an SCI population. The Stigma CAT and SF are readily available for use in both research and clinical settings.

### Suppliers

*Mplus Statistical Analysis with Latent Variables User's Guide* [computer program]. Version 6. Los Angeles: Muthen & Muthen; 2007.

## Disclaimer statements

**Contributors** All authors have contributed significantly to the design, analysis and writing of this manuscript. The contents represent original work and have not been published elsewhere. No commercial party having a direct financial interest in the results of the research supporting this article has or will confer a benefit upon the authors or upon any organization with which the authors are associated.

**Funding** All SCI-QOL items and parameters are © 2015 David Tulsky and Kessler Foundation. All rights reserved. All SCI-QOL items originally from Neuro-QOL are © 2008–2013 David Cella on behalf of the National Institute for Neurological Disorders and Stroke (NINDS). All items are freely available to the public via the Assessment Center platform (www.assessmentcenter.net). There are currently no plans for Dr Tulsky, Kessler Foundation, or the NINDS to profit from the use of the copyrighted material.

**Conflicts of interest** This study was supported by grant #5R01HD054659 from the National Institutes of Health – Eunice Kennedy Shriver National Institute of Child Health and Human Development/National Center on Medical Rehabilitation Research and the National Institute on Neurological Disorders and Stroke.

**Ethics approval** None
